# Association between physical activity practice and sleep quality of older people in social isolation during the COVID-19 pandemic and Health Guidelines and future studies for the post-COVID period: a systematic review

**DOI:** 10.18632/aging.206180

**Published:** 2025-01-15

**Authors:** Alexandro Andrade, Ana Cecília Rosatelli de Freitas Bastos, Anderson D’Oliveira, Guilherme Torres Vilarino

**Affiliations:** 1 Laboratory of Sport and Exercise Psychology, Human Movement Sciences Graduate Program, College of Health and Sport Science of the Santa Catarina State University (UDESC), Florianópolis, Brazil

**Keywords:** exercise, sleep quality, older people, SARS-CoV-2, long COVID

## Abstract

Purpose: Physical activity (PA) is considered an alternative to mitigate the negative impacts of the COVID-19 pandemic on the sleep of older adults. The objective was to verify the association between physical activity and the sleep quality of older people in social isolation during the COVID-19 pandemic, to analyze the Health Guidelines, and suggest future studies for the post-COVID period.

Methods: This systematic review followed PRISMA recommendations, and the protocol was registered in PROSPERO (CRD 42023406471). The search for articles occurred in April 2024 in the databases PubMed, Web of Science, SCOPUS, and gray literature. Data were extracted and checked in a Microsoft Excel^®^ spreadsheet. The quality assessment was performed using tools from the National Institutes of Health.

Results: In total, 1582 studies were found in the databases, of which nine were included in the analyses. Four studies reported a negative association of reduced levels of PA during the pandemic with sleep quality, while one study showed a positive association of PA with sleep quality. Four studies demonstrated no association.

Conclusions: PA was associated with the sleep quality of older adults during the COVID-19 pandemic and reduced levels of PA during this period demonstrated a negative association with sleep quality. Practice of PA is recommended for this post-COVID scenario, as a measure to reduce social isolation and its negative effects and improve the quality of sleep in older adults.

## INTRODUCTION

COVID-19 was declared a pandemic by the WHO on March 11, 2020, due to the significant increases in the number of infected people and the severity of the disease around the world [1]. From that date onwards, social isolation measures were implemented to limit the spread of the virus [2, 3]. Due to the imposed social isolation, the world’s population was exposed to severe changes in lifestyle, including negative changes in nutritional patterns, a reduction in physical activity (PA) levels, and increased loneliness and sleep disorders, among other factors that negatively affected the mental health of the population [2, 4–6].

In this context, the older adult population was considered a risk group, as older age was associated with negative health outcomes related to the impacts of COVID-19 [6, 7], such as hospitalizations and increased chances of death, factors that could be worsened in the presence of comorbidities and low immunity [8, 9]. Furthermore, PA practices underwent negative changes in the COVID-19 period, with a significant decline in PA levels and an increase in sedentary behavior among older adults [6, 10], due to the amplification of various physical, environmental, and psychological barriers, such as difficulty accessing places to practice and lack of motivation, among other obstacles, which needed to be overcome to maintain the practice [11, 12].

Changes in sleep patterns are part of the normal aging process, which is why older people have difficulty falling asleep and staying asleep [13]. Negative changes in sleep quality tend to accompany the evolution of age-related cognitive impairment [14–16]. Furthermore, sleep impairment is related to a reduction in quality of life [17] and the emergence of disorders such as depression [18], considering that this variable can negatively affect emotional regulation [19]. In the context of the pandemic, factors related to isolation, anxiety, fear of being infected, and economic uncertainties negatively affected sleep, promoting changes in the circadian cycle and insomnia [20], increasing sleep problems in the older adult population. During the pandemic period, a 12% increase in insufficient sleep was observed [21], with poor sleep quality in 33.8% of elderly people [22].

In this context, PA practice and sleep quality were considered predictors of changes in mental well-being in different populations, including older adults [23–25]. The practice of PA in conditions of physical and social isolation, in turn, is beneficial and has the potential to alleviate the negative effects of the pandemic on the mental health of this population [6, 26] in addition to being recommended for combating sleep problems [27]; recommendations from health authorities include active breaks during the day, online exercise, and walking [28].

PA can be considered a safe and effective practice to improve sleep quality in older adults due to its well-documented benefits in the literature [29–31]. Considering the deleterious effects of the COVID-19 pandemic on sleep quality and the aggravating factors related to difficulties in maintaining PA in this context, it is important to understand the associations between PA and sleep quality in older adults in social isolation through summarizing evidence on the subject, which can contribute to the identification of the research methods used, gaps in the literature, and the development of new projects in the area. Furthermore, the results found will help health professionals make practical decisions regarding guidance and prescription of PA for the elderly. Therefore, the objective of the current study is to verify the association between the practice of PA and the sleep quality of older adults in social isolation during the COVID-19 pandemic, and to analyze Health Guidelines and suggest future studies for the post-COVID period.

## RESULTS

### Identification and selection of studies

**Figure 1:**
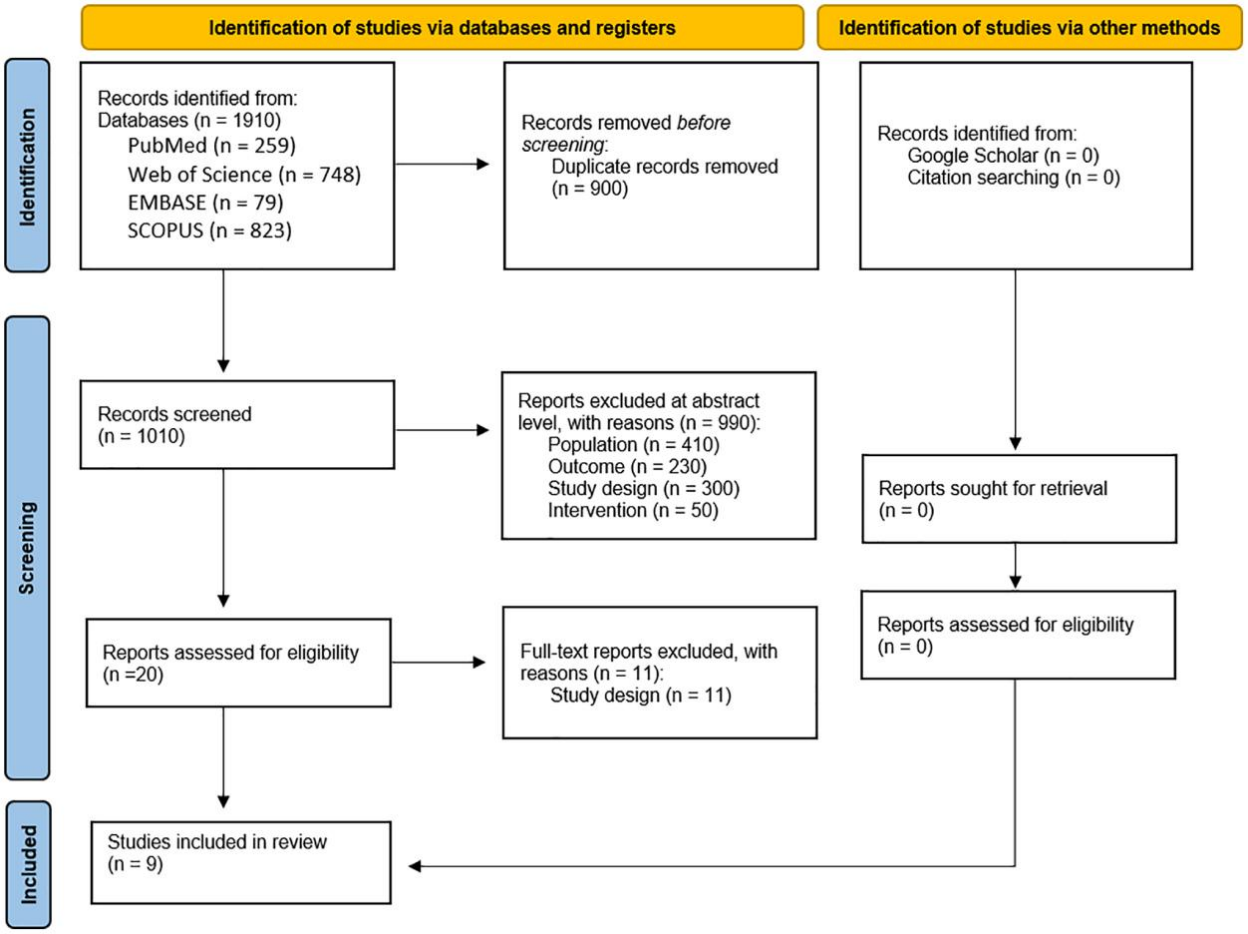
Flow chart of search strategy.

In the first stage of the database search, 1910 articles were found, of which 900 duplicates were excluded and 1010 were selected for reading the title and abstract. At the summary stage, only 20 proceeded to read the full text and of these, only nine met the inclusion criteria and were selected for analysis (Figure 1).

### Characteristics of the included studies

**Figure 2:**
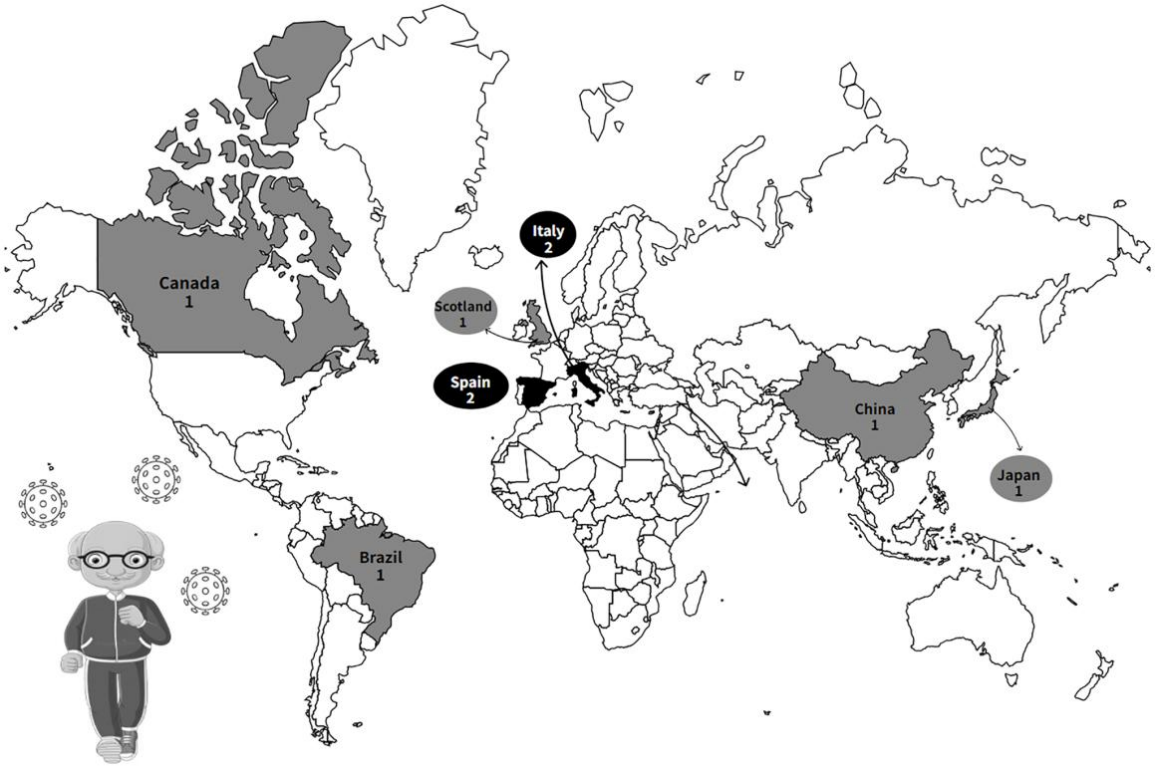
Global geographic distribution of research on the association between the practice of physical activity and the quality of sleep of older adults in social isolation during the COVID-19 pandemic.

Among the nine studies included, the first publications were from 2021 [32–34] and the most recent from 2023 [21, 35, 36]. The studies were conducted in China [37], Japan [32], Brazil [36], Scotland [33], Canada [35], Italy [21, 38] and Spain [34, 39] (Figure 2). The included studies have cross-sectional [21, 32, 36–38] and longitudinal designs [33–35, 39]. The studies analyzed a total of 11,500 older adults of both sexes, aged 60 years or over, who performed different levels of PA.

The studies were carried out between April 2020 [39] and May 2021 [33, 37]. Most studies only evaluated the period “during COVID-19” [32, 35–37], while one study evaluated the period during and “post-COVID-19” [39] and another the “before and during COVID” [21, 33, 34, 38].

### Instruments used to assess sleep

Five studies evaluated sleep quality using the Pittsburgh Sleep Quality Index (PSQI) [21, 33, 36–38]. Two studies evaluated sleep quality through the subjective perception of sleep quality (from very good to very bad) [32, 39]. One study evaluated insomnia using the Insomnia Severity Index (ISI) [35] and one study evaluated the quality of general sleep, obtained from categorization of the results compiled from the various cohort studies that comprised it [34].

### Instruments for assessing PA

The methods used for evaluating PA practice in the studies were diverse, two studies used the short version of the International Physical Activity Questionnaire (IPAQ) [35, 36], one study used the Physical Activity Scale for the Elderly (PASE) [39] and another adopted a compilation of instruments used in the cohort studies that it included, among them: EPIC-cohort questionnaire (EPAQ), Elderly Exernet Physical Activity Questionnaire (EEPAQ), with results expressed as metabolic equivalent (METs), Global Physical Activity Questionnaire, and the Physical Activity Scale for the Elderly (PASE) [34]. In addition to these, the practice of regular or random PA was evaluated according to the score of the Physical Activity Rating Scale (PARS-3). Based on the total score, participants were classified according to the amount and frequency of practice—regular exercise or not [37]. Two studies assessed the average number of hours per week of moderate (e.g., brisk walking, cycling) or intense (e.g., swimming, running) PA [21, 38]. One study assessed regular, or not, physical exercise using the question “Did you practice moderate-intensity exercise (for example, sweating lightly) at least twice a week for 30 minutes over the last year?” classified into regular physical exercise or not [32]. Another study evaluated the question “What level of PA do you practice most?” with responses on a six-point scale ranging from “moving only in connection with necessary (household) tasks” to “keeping fit/heavy exercise or competitive sports several times a week” [33].

### Association between the practice of PA and the sleep of older adults during the pandemic

Six studies reported relationships between PA practice and sleep quality. Among these, three studies found that the decrease in PA levels, when compared to previous periods and during the pandemic, was associated with worsening in sleep quality [21, 34, 38]. In one study, it was found that not complying with the recommendations for PA practice according to the WHO increased the chances of having poor sleep quality [36]. Furthermore, comparing active and inactive older adults, the practice of PA showed a positive association with sleep quality [37].

In four studies there was no association of PA practice with sleep quality [32, 33, 39, 40], and in one study with insomnia [35]. Tables 1 and 2 present the characteristics and results of the studies, as well as the direction of the associations.

**Table 1:** Sample characteristics, study period, pandemic period, study design, sleep measurement, type of PA measurement, and conclusion.

**ID**	**Authors, year**	**Sample characteristics**		**Study period during the pandemic period**	**Period in relation to the pandemic**	**Study design**	**Sleep measurement**	**Type of measurement of AF**	**Conclusion**
		**N/Sex (M–%/F**–**%)**	**Age – *x̄* ± SD (min-max)**						
**1**	Amerio et al. (2023)	4400 (NR)	65 years or older (NR)	November 2020	Before and during	Cross-sectional	Two subscales of the Pittsburgh Sleep Quality Index (PSQI) – Subjective Sleep Quality and Sleep Duration.	The average number of hours per week of moderate (e.g., brisk walking, cycling) or intense (e.g., swimming, running) PA.	The reduction in PA levels during the pandemic was associated with reduced sleep quality.
**2**	Bohn et al. (2023)	1123 (M–10%/F–90%)	67.68 ± 5.92 (NR)	June 2020	During	Cross-sectional	Pittsburgh Sleep Quality Index (PSQI).	International Physical Activity Questionnaire – Short Version (IPAQ-SV) – classification as non-adherent or adherent to PA recommendations (non-adherent: <150 min per week of moderate to vigorous PA; adherent: ≥150 min per week of moderate to vigorous PA).	Failure to comply with *PA guidelines* was negatively associated with sleep quality.
**3**	Gong et al. (2023)	644 (M–26.93%/F–73.07%)	78.73 ± 5.60 (64.20–97.05)	May 2020 - May 2021	During	Longitudinal	Insomnia Severity Index (ISI)	International Physical Activity Questionnaire – Short Version (IPAQ-SV). Moderate and vigorous walking times were evaluated. The metabolic equivalent of the task for the combination of all these activities was calculated.	The practice of PA was not associated with insomnia.
**4**	Stival et al. (2022)	4400 (NR)	65 years or older (NR)	November 2020	Before and during	Cross-sectional	Two subscales of the Pittsburgh Sleep Quality Index (PSQI) – Subjective Sleep Quality and Sleep Duration.	The average number of hours per week of moderate (e.g., brisk walking, cycling) or intense (e.g., swimming, running) PA.	The reduction in PA levels during the pandemic was associated with reduced sleep quality.
**5**	Ju et al. (2022)	568/(M–40.9%/F–59.1%)	NR (60–90)	December 2020–March 2021	During	Cross-sectional	Pittsburgh Sleep Quality Index (PSQI) – rating on good sleep quality or not.	*Physical Activity Rating Scale* (PARS-3): based on the total score, the participants were classified according to the amount and frequency of practice – classification in regular PA practice or not.	More older adults who practiced regular PA reported better sleep quality when compared to non-practitioners.
**6**	Rodrigues-Gómez et al. (2022)	1092/(M–33.5%/F–66.5%)	80.3 ± 5.6 (NR)	April-December 2020	During and after	Longitudinal	Subjective sleep quality (Very good, good, normal, poor, very poor).	*Physical Activity Scale for the Elderly* (PASE) - classification of the PA level based on the tertiles of the group, with those with the lowest PA being at T1 and those with the highest PA being at T3/NR.	The practice of PA was not associated with the quality of sleep in the older adults.
**7**	García-Esquinas et al. (2021)	3041/M/F (NR%)	74.5 (NR)	May-June 2020	Before and during	Longitudinal	Sleep Quality.	*EPIC-cohort questionnaire (EPAQ), Elderly EXERNET Physical Activity Questionnaire (EEPAQ),* with results expressed as metabolic equivalent (METs), *Global Physical Activity Questionnaire,* and a *Physical Activity Scale for the Elderly (PASE).*	The reduction in PA levels during the pandemic was associated with reduced sleep quality.
**8**	Makizako et al. (2021)	178/(M–46%/F–54%)	69.7 ± 4.2 (NR)	February 2021	During	Cross-sectional	Subjective sleep quality (Very good, good, poor, and very poor).	Regular PA practice at least 2 times a week lasting thirty minutes or more for at least 1 year – classification into regular PA habit or not.	The practice of regular PA was not associated with sleep quality.
**9**	Okely et al. (2021)	454/(NR)	84	May 2021	Before and during	Longitudinal	Single question adapted from the Pittsburgh Sleep Quality Index (PSQI) “During the last month, how would you rate your overall sleep quality?” (Very good, good, bad and very bad).	Questionnaire - “What level of physical activity do you do the most?” Response on a six-point scale ranging from “moving only in connection with necessary (house) chores” to “keeping fit/heavy exercise or competitive sports several times a week.” Responses were scored from 1 to 6, with a higher score indicating more PA.	PA practice was not associated with sleep quality.

Abbreviations: EEPAQ: Elderly EXERNET Physical Activity Questionnaire; EPAQ: EPIC-cohort questionnaire; F: Female; IPAQ-SV: International Physical Activity Questionnaire Short Version; ISI: Insomnia Severity Index; M: Male; METS: Metabolic Equivalent; N: Number of participants in the sample; NR: Does Not Report; PA: Physical Activity; PARS-3: Physical Activity Rating Scale; PASE: Physical Activity Scale for the Elderly; PE: Physical Exercise; PSQI: Pittsburgh Sleep Quality Index; SD: Standard Deviation; T1: first tertile; T3: third tertile.

**Table 2:** Main results of studies on the associations between PA practice and sleep quality.

**ID**	**Authors, year**	***N***	**Findings**	**Association - + More PA better SQ**	**Association – Less PA, worse SQ**
**1**	Amerio et al. (2023)	440	The reduction in PA levels during the pandemic was associated with reduced sleep quality (OR 2.05 (1.67–2.53)).	↑	↓
**2**	Bohn et al. (2023)	1123	Failure to comply with PA recommendations was negatively associated with sleep quality (OR 0.637; *p* = 0.00).	↑	↓
**3**	Gong et al. (2023)	644	The practice of PA was not associated with insomnia.	—	—
**4**	Stival et al. (2022)	4440	Reduced PA levels during the pandemic were associated with reduced sleep quality (OR 2.45 (1.91–3.15)).	↑	↓
**5**	Ju et al. (2022)	568	Older adults who practiced regular PA reported better sleep quality when compared to non-practitioners.	↑	↑
**6**	Rodrigues-Gómez et al. (2022)	1092	The practice of PA was not associated with the quality of sleep in the older adults.	—	—
**7**	Garcia-Esquinas et al. (2021)	3041	The reduction in PA levels during the pandemic was associated with reduced sleep quality.	↑	↓
**8**	Makizako et al. (2021)	178	Regular PA was not associated with sleep quality (OR 0.76 (0.32–1.80)).	—	—
**9**	Okely et al. (2021)	454	PA practice was not associated with sleep quality.	—	—

Note: ↑ - Association of PA practice with sleep quality; ↓ - Association of reduced PA or physical inactivity with sleep quality; — - There was no association between PA practice and sleep quality. Abbreviations: PA: Physical activity; SQ: sleep quality.

#### Assessment of the quality of the studies

**Figure 3:**
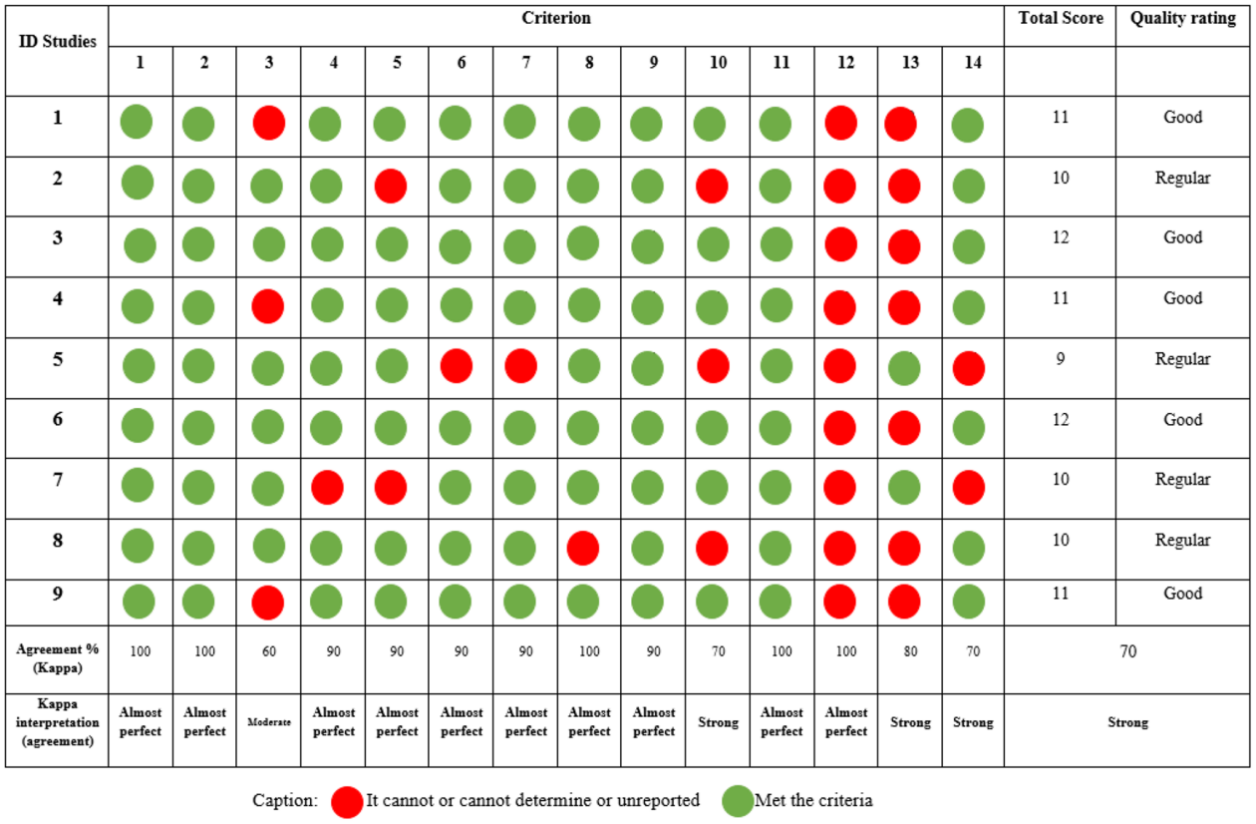
Evaluation of the quality of observational studies.

Figure 3 presents the general and specific classifications of the criteria for evaluating the quality of studies [41]. The observational studies included scored an average of 10.7 (±0.94), with a range of 9 to 12. Based on this assessment, the quality was considered good in five studies and fair in four. Agreement between observers for all items was 70% overall (Figure 3).

Information about the blinding of outcome assessors regarding participant exposure was not reported in any observational studies, which could lead to confounding factors. Well-conducted observational studies control for multiple potential confounders. On the other hand, it was observed that researchers had significant concerns regarding the use of appropriate tools and methods to measure the variables of interest investigated, and considering whether exposure measurements were accurate and reliable (criteria 9 and 11), all studies reported affirmative information, which directly influences confidence in the reported exposures and their results.

## DISCUSSION

During the COVID-19 pandemic, the importance of PA was verified as a protective factor for the physical and mental health of the older adult population [6, 42]. On the other hand, sleep disorders became more common during this period, also affecting the older adult population [43]. The reductions in PA levels caused by the pandemic are related to negative changes in sleep quality, and in this context, the practice of PA in isolation conditions has been identified as an alternative to mitigate these negative effects [44–46]. Therefore, the objective of the current review was to investigate the association between the practice of PA and the sleep quality of older adults in social isolation during the COVID-19 pandemic. In general, physical inactivity had a negative association with sleep quality [21, 34, 36–38]. The discussion of the results presented in the articles included in this review will be performed according to the associations found.

### Association between reduced physical activity and sleep quality in the older adults

Four of the studies analyzed demonstrated negative effects of reducing physical activity levels on sleep quality during the pandemic [21, 34, 36, 38] of which three studies analyzed PA levels between the previous period and during the COVID-19 pandemic [21, 34, 38]. PA deprivation is relevant, especially considering the bidirectional relationship between PA and sleep, which may have led to a feedback loop of physical inactivity and poor sleep quality during this period [47], factors that have been considered predictors of mental well-being during the pandemic period [48]. Despite this, the study by Diniz et al. [49] observed similar results during this period, in which insufficient activity levels were considered risk factors for sleep pattern disorders in the general population [49].

The study by Bohn et al. [36], analyzed the influence of the rigidity of confinement and the practice of PA and other factors on the sleep of older adults [36]. The authors found that failure to meet PA recommendations had a negative association with sleep quality, perceived quality of life, and depressive symptoms. These results demonstrate that during the pandemic period, physical inactivity directly impacted the quality of sleep of older adults, which may have negatively affected quality of life and depression, considering that sleep quality has already demonstrated an association with these factors in previous studies [50, 51], and that physical inactivity can amplify the relationships between sleep quality and depressive symptoms [52].

It is essential to highlight that when verifying that reduced levels of PA are related to worsening sleep quality in older adults, the findings described in the studies actually strengthen the hypothesis of the positive effects of PA on the health of this population and the production of scientific research demonstrating positive effects of PA on the sleep of older people.

### Association between PA and sleep in older adults

In the study by Ju et al. [37], it was observed that older adults who practiced PA regularly reported better sleep quality when compared to those who did not practice PA. These results corroborate the literature, which demonstrates that regular PA can lead to improved sleep quality, reduced sleep latency, and better overall sleep quality [47]. Nevertheless, PA is related to the stability of the circadian rhythm and well-being [48].

While general PA may be interesting for improving sleep quality [47], PA interventions [31] and cognitive behavioral therapy [53] are strategies previously studied in the older adult population to reduce sleep disorders. The literature points to significant benefits of aerobic exercise for sleep quality, such as reducing REM sleep and increasing restful sleep. [50]. An eight-week progressive web-based Hatha Yoga intervention improved the overall sleep quality of older adults during the pandemic [51]. However, intervention studies in this period are still scarce, considering that the continuity of structured PA interventions during the isolation period of the COVID-19 pandemic was hampered due to the increase in barriers to the practice [11]. This factor was evidenced in the study by Miller et al. [52], in which the authors reported the intention to continue the intervention started in 2019, however, the conditions of the pandemic did not allow it.

### The lack of association between PA and sleep in older adults

Four studies included in the review did not find associations of PA practices with sleep quality in older adults [32, 33, 39, 40], and one study with insomnia [35]. These results contrast with the main findings currently debated in the literature, which reveal relevant associations between PA and sleep [54].

While most studies that presented some type of association between PA and sleep quality were collected via telephone or in person [21, 34, 36–38], of the studies that did not show an association, two applied the questionnaires via telephone and two online. When collecting data, online methods may present a significant bias, considering that although the use of technology has increased significantly in this population [55], barriers such as configuration difficulties, confusing instructions, the need for help from other people, and frustration are reported by older adults concerning the use of smartphones or tablets, which can influence the interpretations and answers to the questionnaires [56]. Despite this, several studies have opted for online data collection and interventions [57, 58], finding no differences compared to studies carried out in person [58].

It is known that over time the cognitive process of older adults deteriorates, making it necessary in scientific research to use instruments that assess cognitive capacity to mitigate possible biases in the interpretation of questions and applications of instruments [59]. Only subjective measures were used for the assessments and only two of the nine included studies assessed the cognitive function of the older adults [34, 35]. In addition, although the PSQI is the most widely used instrument for measuring sleep due to its low cost and easy application [31], its interpretation may suffer from biases related to the cognitive capacity of older adults and differences between objective and perceived sleep quality [60]. A similar scenario is observed in the subjective measurement of PA practice, in which older adults tend to overestimate their levels of practice [61] and the cognitive status of the older adults can accentuate this overestimation [62]. Therefore, studies have highlighted the relevance of the association between objective sleep measures, such as actigraphy, and the use of a questionnaire to obtain more accurate results on sleep quality, as well as the use of objective measures of PA, aiming to reduce biases related to the instruments used [31, 60–62].

Furthermore, considering the diversity of countries in the studies analyzed, cultural and socioeconomic differences between the samples, related to PA practice patterns and sleep quality, may have influenced the results. Older adults from countries with low socioeconomic status tend to perceive more barriers to practicing PA [63] and present lower levels of sleep quality [64], especially compared to those with higher socioeconomic status [65]. In both studies carried out in developing countries (Brazil and China), relevant associations were observed between physical inactivity [36], and PA and sleep quality [37].

It is worth mentioning that the methods adopted for assessing PA and sleep quality were diverse. PA was measured in seven different ways, with specific scales for the elderly (PASE) [34, 39], the IPAQ short version [35, 36], and reports of the number of weekly hours of PA [21, 38], among others. To measure sleep, one to two questions were used [21, 33, 38] or the full PSQI questionnaire [36, 37], subjective sleep quality scales [32, 34, 39] and the insomnia severity questionnaire [35]. Therefore, considering the diversity of instruments used to assess the exposure and outcome, comparisons between study results tend to be difficult. Furthermore, the study sample was predominantly female, with low male participation, making it difficult to extrapolate the results presented for this population.

### Health guidelines and perspectives for future studies in the post-COVID period

**Figure 4:**
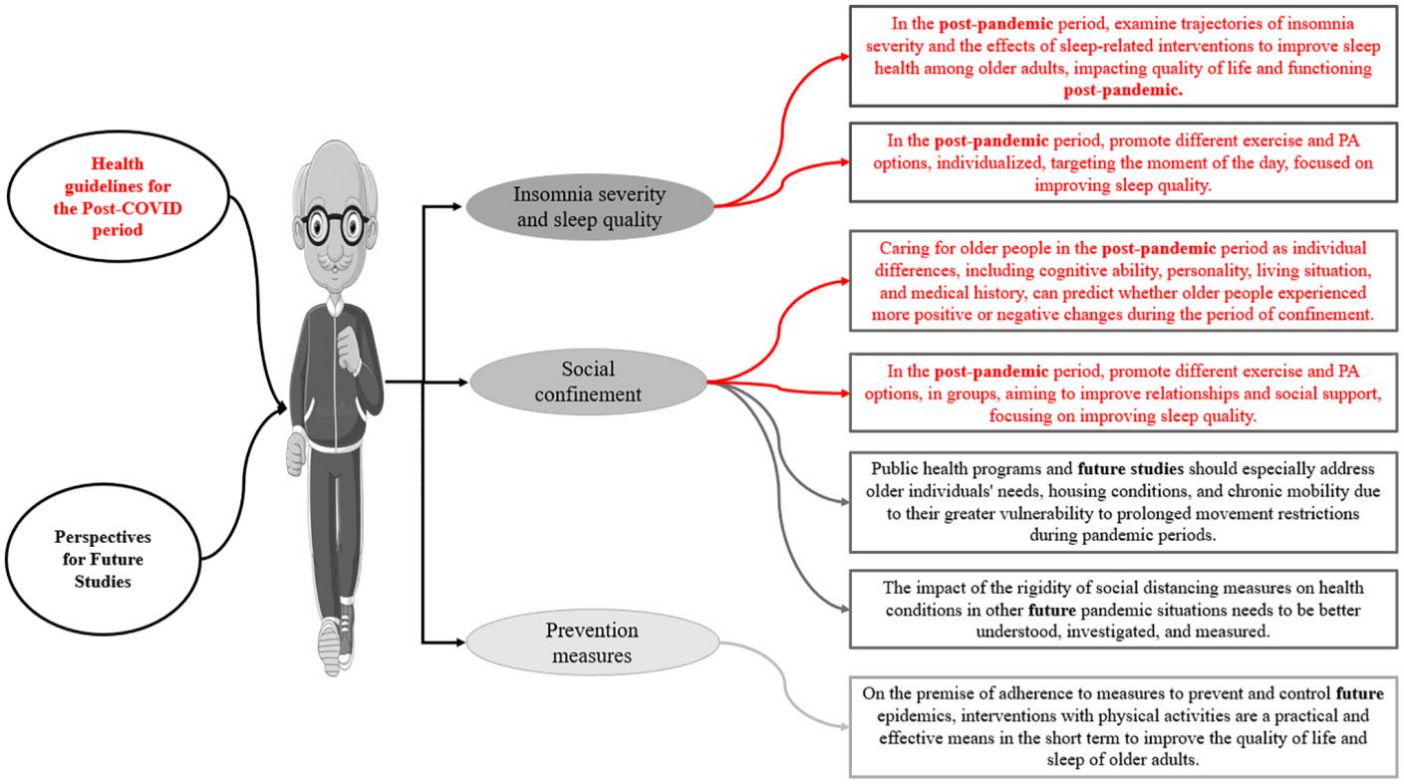
Health guidelines and perspectives for future studies in the post-COVID period.

Based on the recommendations of the studies included in this review, we stratified relevant information for intervention measures for the health of older adults, related to PA and exercise and sleep quality. These measures are aimed at public policymakers, health professionals, and researchers, to develop new public policies based on evidence, as well as identifying gaps to be addressed in future research. Guidelines for the health of older adults were highlighted for the post-COVID-19 pandemic period, since the pandemic period brought harm due to social isolation and the consequences of the disease on human health. Based on these points, the precautions and observations that need to be taken into consideration are presented in Figure 4, highlighting measures to improve sleep quality, and reduce social confinement, including exercise and PA, as well as recommendations for future studies.

#### Clinical implications

The results found present important clinical implications that will help health professionals in their decision-making. The recommendation for the practice of aerobic or anaerobic PA should be encouraged.

Although the authors evaluated PA practices in general terms, not focusing specifically on the modalities, but rather on time and intensity, it is understood that the use of the practice regardless of the modality, but alternating between moderate and vigorous use, may present positive associations in the regulation of sleep in older adults both in a period of global pandemic and throughout their lives, as well as in the new normal post-COVID-19.

Older adults affected by COVID-19 sequelae can benefit from treatment through PA practices as quickly as possible as a way of reducing the time spent suffering from various complications and reducing the demands on health professionals and caregivers [66, 67].

#### Limitations and future studies

The current review presents limitations related to the number of studies included, the methodological differences between them, and the quality of the studies. The study methods regarding exposure to PA and sleep analysis differ significantly, making it difficult to compare results. To assess PA, nine different scales were used, however, only two studies used a specific scale for the older adult population, the PASE [34, 39]. Regarding sleep collection, there were also divergences, six studies used the PSQI, either with only some subscales or the full version [21, 33, 36–38], three studies evaluated the subjective sleep quality [32, 34, 39] and one study evaluated insomnia [35].

Another issue is that other diseases that could interfere with the quality of sleep in the elderly were not considered, as it is common for them to present changes in their health and this can increase sleep disorders [5, 68].

The studies were conducted in a few countries, highlighting the need to produce more studies in different countries, to obtain a more representative global panorama and in-depth analyses concerning the socioeconomic status of each country.

Therefore, future studies should consider evaluating associations between PA and sleep quality, prioritizing the use of face-to-face or telephone interviews, with cognitive assessment of the older adults, using direct and indirect methods to assess variables, and prioritizing gold standard instruments developed especially for this purpose and population, evaluating different facets of the variables analyzed, considering increasing the methodological quality, avoiding even more biases. It is essential to develop efforts for studies that consider the post-COVID and long-COVID scenario, monitoring older people who were affected by different levels of symptoms after COVID-19 infection, related to different ways of practicing PA and the quality of sleep.

#### Strength, innovations and applications

This is the first study to investigate the associations between PA practice and the quality of sleep of older adults during the COVID-19 pandemic, seeking to understand the factors related to these associations, as well as possible biases in the studies. It is important to highlight that all studies included in this review were of good to fair quality. This result is related to the authors’ lack of reporting on the blinding of outcome assessors and appropriate statistical procedures to minimize confounding factors. Methodologically well-conducted studies allow for more precise generalizations. However, the quality of the included studies does not allow generalizations, and their results must be interpreted concisely and with caution. Therefore, the current study is relevant because it brings together the available literature on the association between the practice of PA and the quality of sleep of older adults during the pandemic and despite the methodological diversity between the studies, the majority presented good quality. Through this review, it was possible to highlight how the reduction in PA levels is associated with a reduction in the quality of sleep in older adults, therefore, it serves as a warning that in future pandemics, actions need to be taken to maintain PA levels or to promote PA.

The current study presented a general overview of the distribution of scientific production in the area of PA and quality of sleep in older adults in the context of social isolation.

The main applications of the findings of this study are related to the best hypothesis of the positive effects of exercising and physical activities on the physical and psychological health of older people in general, specifically the positive effects on improving the quality of sleep and socialization of this population are well documented. Applying exercise and encouraging the practice of physical activities based on this foundation, strengthened by the findings of our study, is consistent with the concern regarding the health of older people in this post-COVID scenario. These applications seek to avoid the damage caused by the reduction or deprivation of physical activity in the context of social isolation, unfortunately, common in the lives of many older people even outside the pandemic scenario.

## CONCLUSIONS

Based on the analysis of the results of the selected articles, we found that PA may be associated with the sleep quality of older adults during the COVID-19 pandemic and that reduced levels of PA during the COVID-19 pandemic period had a negative association with the quality of sleep of older adults in social isolation. The practice of exercise and PA, individually and in groups, is recommended for this post-COVID scenario, as a measure to reduce social isolation and its negative effects and improve the quality of sleep in older adults.

Future studies should consider evaluating associations between PA and sleep quality, evaluating older adults in person or by telephone, trying to control as many confounding variables as possible, and prioritizing gold-standard instruments specifically for older adults.

## MATERIALS AND METHODS

This systematic review followed the recommendations of the Preferred Reporting Items for Systematic Reviews and Meta-Analyses (PRISMA) [69] and was registered in the International Prospective Register of Systematic Reviews (PROSPERO) – CRD 42023406471.

### Search strategy

The search was conducted by two researchers (ACR and AD), independently, in the databases: PubMed, Web of Science, Embase, SCOPUS and Google Scholar for gray literature in April 2024. The primary and secondary search terms for the studies were included in the title, abstract, and keywords fields of each database and all descriptors used are organized in Table 3.

**Table 3:** Search strategy for the systematic review.

**Database Search**	
**Terms**	**Descriptors**
**Elderly**	Elderly OR Aged OR Aging OR “Aged, 80 and over” OR “older adults” OR “older women” OR “older men” OR Senescence OR “Oldest Old” OR “Old Adults” OR Nonagenarian* OR Octogenarian* OR Centenarian*.
**Sleep**	Sleep OR “Sleeping Habits” OR “Sleep Habits” OR “Habit, Sleep” OR “Habits, Sleep” OR “Sleep Habit” OR “Sleeping Habit” OR “Habit, Sleeping” OR “Habits, Sleeping” OR “sleep quality” OR “sleep disturbances”.
**Physical activity**	“physical activity” OR “exercise” OR “physical exercise*” OR “sport*” OR “muscle stretching exercises” OR “resistance training” OR “exercise isometric” OR “exercise movement technique*” OR “exercise therap*” OR “isometric exercise*” OR “muscle stretching exercise*” OR “strength training” OR “strength training program*” OR “training resistance” OR “weight-bearing exercise” OR “weight bearing strengthening program” OR “weight bearing” OR “weight lifting exercise” OR “weight lifting” OR “hydrotherapy” OR “aquatic exercise” OR “water exercise” OR “balance exercise”.
**COVID-19**	“2019 novel coronavirus disease” OR “COVID-19” OR “COVID-19 pandemic” OR “SARS-CoV-2 infection” OR “COVID-19 virus disease” OR “2019 novel coronavirus infection” OR “2019-nCoV infection” OR “coronavirus disease 2019” OR “coronavirus disease-19” OR “2019-nCoV disease” OR “COVID-19 virus infection”.

The Web of Science database was prioritized when deciding on duplicate articles. The searches were carried out in the Main Collection in the basic research field with terms related to older adults, sleep quality, PA, and COVID-19 in “topic”, and the time stipulated as “every year”. Additionally, the authors searched the reference lists of all identified studies to find other relevant articles [70].

### Eligibility criteria

This systematic review included cross-sectional and longitudinal studies with older adults (≥60 years old), of both sexes, healthy or with associated comorbidities, which evaluated the practice of PA on the quality of sleep of older adults during the COVID-19 pandemic. The eligibility criteria were based on the PECOS strategy: Population, Exposure, Comparison, Outcomes, and Study Design [71] and are described in Table 4.

**Table 4:** Criteria for inclusion and exclusion of studies selected for the review.

		**Inclusion criteria**	**Exclusion criteria**
**P**	Participants	Older adults aged 60 and over.	Only obese older adults with chronic diseases or Parkinson’s.
**E**	Exposure	Physical activity, resistance exercises, aerobic exercises, aquatic exercises, stretching exercises, balance exercises, and sports.	−
**C**	Comparison	−	−
**O**	Outcomes	Association of physical activity and sleep quality in older adults during the COVID-19 pandemic.	Studies that evaluated only sleep duration.
**S**	Study Design	Cross-sectional and longitudinal studies.	

### Study selection

The selection of studies was carried out independently by two researchers (ACR and AD). In case of disagreement between the researchers, a third evaluator (AA) was responsible for giving their opinion for the final decision. The authors examined all titles and abstracts and reviewed the full texts of the articles that met the predetermined inclusion and exclusion criteria. All these steps were performed on Microsoft Excel spreadsheets, from the export of results from each database to the selection of studies that were part of the review. References in the included articles were reviewed to identify other potentially relevant studies.

### Data extraction

For synthesis and discussion of results, data on the study design and authors, journal, country/city where the study was conducted, sample, sex and age of the sample, period during which the study was carried out, sample characteristics, outcome variable investigated, period of studies concerning COVID-19 (before, during, after), type of PA, type of PA measurement, main findings, and conclusion were extracted.

### Risk of bias

The quality of included studies was assessed using the tool - Quality Assessment Tool for Observational Cohort and Cross-Sectional Studies [41]. The tool contains criteria that need to be met by the studies and at the end of the evaluation a score is assigned according to each criterion answered; one point for a “yes” answer and zero points for “no”, “not applicable”, “not reported” or “cannot determine”. The Cohort and Cross-sectional Studies tool has an assessment based on 14 criteria. A study-specific global score, ranging from zero to 14, was calculated by summing scores across all criteria. The quality of the studies was classified as poor (0–4 in 14 questions), regular (5–10 in 14 questions) or good (11–14 in 14 questions). A higher score indicates a better quality of study [41]. Previous studies used these tools demonstrating satisfactory applicability [72, 73].

Two researchers (ACR and AD) independently performed the risk of bias assessment and disagreements were resolved by consulting a third reviewer (GTV). The degree of agreement between the two evaluators was analyzed using Cohen’s Kappa test, interpreted according to McHugh’s approach [74], which classifies agreement as: None 0–4%; minimum 4–15%; weak 15–35%; moderate 35–63%; strong 64–81%; and near perfect 82–100%.
